# Alterations in Peripheral Blood B Cell Subsets and Dynamics of B Cell Responses during Human Schistosomiasis

**DOI:** 10.1371/journal.pntd.0002094

**Published:** 2013-03-07

**Authors:** Lucja A. Labuda, Ulysse Ateba-Ngoa, Eliane Ngoune Feugap, Jorn J. Heeringa, Luciën E. P. M. van der Vlugt, Regina B. A. Pires, Ludovic Mewono, Peter G. Kremsner, Menno C. van Zelm, Ayola A. Adegnika, Maria Yazdanbakhsh, Hermelijn H. Smits

**Affiliations:** 1 Department of Parasitology, Leiden University Medical Center, Leiden, The Netherlands; 2 Centre de Recherches Médicales de Lambaréné (CERMEL), Lambaréné, Gabon; 3 Institute of Tropical Medicine, University of Tübingen, Tübingen, Germany; 4 Department of Immunology, Erasmus MC, Rotterdam, The Netherlands; Uniformed Services University of the Health Sciences, United States of America

## Abstract

Antibody responses are thought to play an important role in control of *Schistosoma* infections, yet little is known about the phenotype and function of B cells in human schistosomiasis. We set out to characterize B cell subsets and B cell responses to B cell receptor and Toll-like receptor 9 stimulation in Gabonese schoolchildren with *Schistosoma haematobium* infection. Frequencies of memory B cell (MBC) subsets were increased, whereas naive B cell frequencies were reduced in the schistosome-infected group. At the functional level, isolated B cells from schistosome-infected children showed higher expression of the activation marker CD23 upon stimulation, but lower proliferation and TNF-α production. Importantly, 6-months after 3 rounds of praziquantel treatment, frequencies of naive B cells were increased, MBC frequencies were decreased and with the exception of TNF-α production, B cell responsiveness was restored to what was seen in uninfected children. These data show that *S. haematobium* infection leads to significant changes in the B cell compartment, both at the phenotypic and functional level.

## Author Summary

Schistosomiasis affects over 200 million people and especially children in developing countries. It causes general hyporesponsiveness of the immune system, which until now has predominantly been described for various T cell subsets as well as dendritic cells. B cells in this context have not yet been investigated. To address this question, we phenotyped B cell subsets present in peripheral blood from *S. haematobium* infected and uninfected schoolchildren living in an endemic area in Lambaréné, Gabon. Children with schistosomiasis had an increased frequency of various memory B cell subsets, including subsets associated with B cell exhaustion, and a concomitant decrease in naive B cells. To study the effect of *Schistosoma* infection on B cells in more detail we isolated peripheral blood B cells and found that B cells from infected children had a reduced capacity to proliferate and produce TNF-α in response to both B cell receptor and Toll-like receptor stimulation. These results provide new insights into the role of B cells in the host immune response to schistosomiasis and may provide a novel target for therapeutic strategies.

## Introduction

Schistosomiasis is a major parasitic disease of humans in the developing world, with over 200 million people infected worldwide [1]. As with other chronic helminth infections, schistosomes cause widespread immune activation and deregulation leading to general T cell hyporesponsiveness supporting the long term survival of the parasite and minimizing immunopathology [2]–[4]. Resistance to schistosomiasis is only gradually acquired and is attributed to cumulative exposure to infection [5], [6]. Mice vaccination experiments with radiation-attenuated *S. mansoni* cercariae showed less protection against re-infection in µMT B cell-deficient mice than in wild-type mice [7], and the transfer of serum from infected rodents to naive animals can protect against infection [8], [9], suggesting that antibodies are important for protection against infection. In human infection, protective IgA, IgE and IgG levels have been demonstrated against adult worm antigens [10], [11], and resistance to (re-) infection is correlated with an increased ratio between IgE and IgG4 [12]. Furthermore, expression of CD23, the low affinity IgE receptor which can be strongly up-regulated by IL-4 [13], is also correlated with development of resistance to *Schistosoma* re-infection [14], [15]. While B lymphocytes support the establishment of the strong Th2 profile associated with helminth infections [16], more recently they have also been shown to play an active regulatory role in the course of *Schistosoma* infections [17] mostly effecting T cell responses.

In general, immunological memory is characterized by its ability to respond more rapidly and robustly to re-infection and is dependent on the generation and maintenance of memory B cells (MBCs) [18]. Memory B cells, originally defined as CD27^+^ [19], can be further characterized into additional subsets by co-staining with IgD into non-switched MBCs (CD27^+^IgD^+^), switched MBCs (CD27^+^IgD^−^) and double negative MBCs (CD27^−^IgD^−^) [20]. Furthermore, co-staining with CD21 can be used to separate classical MBCs (CD27^+^CD21^+^) from activated MBCs (CD27^+^CD21^−^) and atypical MBCs (CD27^−^CD21^−^) [21]. Based on these markers, naive B cells can be classified as CD27^−^IgD^+^or CD27^−^CD21^+^. Recent studies have shown that chronic HIV infection [21], [22] as well as exposure to and infection with *P. falciparum* malaria [23], [24] are associated with the expansion of atypical or ‘exhausted’ MBCs (CD27^−^CD21^−^). These cells are characterized by high expression of the inhibitory receptor FCRL4 [25], [26], and it has been suggested that this population may contribute to diminished pathogen-specific antibody responses in infected individuals. Other chronic infections such as hepatitis C virus (HCV) [27] have also shown perturbations in the distribution of peripheral B cells subsets, most notably within the memory B cell compartment suggesting that MBCs may play a role in disease pathogenesis as well as insufficient immune response to combat the disease.

Ligation of the B cell receptor (BCR) by its cognate antigen leads to the production of antibodies and, depending on the cytokines produced by Th cells, to further antibody isotype switching and affinity maturation. B cells can also express a variety of innate receptors, most notably Toll-like receptors (TLRs), and can play a significant role in innate immune responses as B cells upregulate activation markers, proliferate and secrete cytokines upon engagement of these receptors [28], [29]. Importantly, TLR stimulation can also potentiate the T cell-dependent production of antibodies [30], [31]. TLR9 is highly expressed in human B cells and is ligated by bacterial DNA motifs containing unmethylated CpG dinucleotides. Previous studies have clearly demonstrated that TLR9 stimulation is sufficient to directly induce both naive and memory B cell proliferation and activation [32], [33]. In addition, the role of B cells in innate immune responses has gained further interest as several studies have demonstrated a pathogenic role for B cells independent of their ability to produce antibodies. For example, systemic lupus erythematosus (SLE) and rheumatoid arthritis (RA) are associated with abnormally increased pro-inflammatory cytokine production by B cells, including TNF-α. Importantly TNF-α [34], along with more recently IL-17 [35], is also one of the key cytokines involved in schistosome-induced pathology [36], [37].

Currently there is little information on the composition of the peripheral blood B cell compartment or the concomitant adaptive and innate functionality of B cells during the course of human *Schistosoma haematobium* infection. In this study, we compared the circulating B cell subsets in peripheral blood of schistosome-infected and uninfected Gabonese schoolchildren and their B cell response to BCR and TLR engagement.

## Materials and Methods

### Study population

In April 2008 we initiated a study to investigate the effect of *S. haematobium* infection on B cell phenotype and function. Venous heparinized blood was obtained from 56 school-aged children living in Lambaréné (Gabon), a semi-urban municipality or from its surrounding villages in which *Schistosoma haematobium* infection is endemic and has been previously described in detail [17], [38], [39]. *S. haematobium* infection was determined prior to blood collection by examining a filtrate of 10 ml of urine passed through a 12-µm-poresize filter (Millipore). Children were classified *S. haematobium*-infected if at least one *S. haematobium* egg was detected in the urine, or uninfected if three consecutive urine samples were negative. Infections with intestinal helminths *Ascaris lumbricoides* and *Trichuris trichiura* were determined by analyzing one fresh stool sample using the Kato-Katz method [40]. Hookworm larvae were determined in a 7-day coproculture of the same stool sample [41]. Infection with *Plasmodium falciparum* was determined by Giemsa-stained thick blood smears [42]. After collection of blood samples, all *S. haematobium*-infected children were treated with a single dose of praziquantel (40 mg/kg) three times every two months. Intestinal helminth- and malaria-infected children received respectively a single dose of albendazole (400 mg) and an artemisinin-based combination therapy as per the local guidelines. The study was approved by the “Comité d'Ethique Regional Independent de Lambaréné” (CERIL; N°06/08). Written informed consent was obtained from parents or legal guardians of all schoolchildren participating in the study.

### Cell isolation

PBMCs were isolated by Ficoll-Hypaque density gradient centrifugation from 20 ml of heparinized blood. B cells were isolated with anti-CD19 MicroBeads (Miltenyi Biotec). The isolated B cells were routinely ∼95% pure.

### Immunoglobulin assays

Plasma samples were analyzed using the Bio-Plex Pro Assays Immunoglobulin Isotyping Kit (Bio-Rad) for total IgM, IgG1, IgG2, IgG3, IgG4 and IgA levels according to manufacturers' recommendations. Levels of total IgE were measured by ELISA according to manufacturers' instructions (Allergopharma).

### B cell stimulation, staining of CD23 and intracellular TNF-α and Ki-67

Freshly isolated B cells were cultured in RPMI 1640 medium (Invitrogen) supplemented with 10% FCS (Greiner Bio-One), 100 U/ml penicillin (Astellas), 10 µg/ml streptomycin, 1 mM pyruvate and 2 mM L-glutamine (all from Sigma). B cells were seeded at 1×10^5^ cells per well and stimulated for 48 hours with 2.5 µg/ml anti-human IgG+IgM (Jackson ImmunoResearch), 5 µg/ml CpG ODN 2006 (Invivogen) or anti-IgG/IgM+CpG. To detect intracellular TNF-α, B cells were restimulated with 50 ng/ml PMA (Sigma-Aldrich), 2 µg/ml ionomycin (Sigma-Aldrich), and 100 ng/ml ultrapure LPS (Invivogen) for 6 hours with the final 4 hours in the presence of 10 µg/ml brefeldin A (Sigma-Aldrich), followed by fixation with the FoxP3 fixation/permeabilization kit (eBioscience) and frozen in RPMI supplemented with 20% FCS and 10% DMSO (Merck) and stored at −80°C until FACS analysis. After thawing, cells were permeabilized (Permeabilization buffer, eBioscience) and labeled with surface anti-CD10-PerCP/eF710 (SN5c, eBioscience), anti-CD20-APC/eF780 (2H7, eBioscience), anti-CD21-FITC (LT21, BioLegend), anti-CD23-PE/Cy7 (EBVCS2, eBioscience), anti-CD27-APC (L128, BD Biosciences), and intracellular anti-Ki-67-eF450 (20Raj1, eBioscience) and anti-TNF-α-biotin (MAB11, eBioscience) followed by second incubation with streptavidin-Qdot525 (Invitrogen).

### Characterization of B cells in peripheral blood

For immunophenotyping freshly isolated PBMCs were fixed in 2.4% formaldehyde (Sigma-Aldrich) for 15 minutes at room temperature and, subsequently, frozen in RPMI 1640 medium supplemented with 20% FCS and 10% DMSO and stored at −80°C until FACS analysis. After thawing, cells were washed and stained for 30 minutes with anti-CD19-PB (HIB19, eBioscience), anti-CD21-FITC (LT21, BioLegend), anti-CD27-APC (L128, BD Biosciences), anti-CD27-APC/eF780 (O323, eBioscience), anti-HLA-DR-APC/Cy7 (L243, BioLegend) and anti-IgD-biotin (IA6-2, BD Biosciences) followed by second incubation with streptavidin-Qdot525 (Invitrogen). Anti-FCRL4-biotin was kindly provided by M. Cooper (Emory University School of Medicine, Atlanta, GA, USA). Alternatively, cells were stained with anti-CD19-BV510 (HIB19, BioLegend), anti-IgA-FITC, anti-IgA-PE (both IS11-8E10, Miltenyi Biotec), anti-CD21-PE/Cy7 (LT21), anti-CD23-APC (EDVCS5), anti-CD27-PerCP/Cy5.5 (L128), anti-CD38-APC/Cy7 (HB7), anti-IgD-PE/CF594 (IA6-2), anti-IgG-PE (G18-145) and anti-IgM-V450 (G20-127) (all from BD Biosciences). Cells were acquired on FACSCanto II and LSR Fortessa flow cytometers (both from BD Biosciences).

### Statistical analysis

Differences between study groups were tested using Fisher's exact test for gender and co-infections and by Mann-Whitney U test for *Schistosoma* infection intensity. Age was normally distributed and differences between infection groups were tested using the independent student's T test. Serological and cellular differences between infection groups were tested by the Mann-Whitney U test. Differences within the same group between baseline and follow-up were compared by Wilcoxon matched pairs test. P-values less than 0.05 were considered significant and less than 0.1 a trend. *** p<0.001, ** p<0.01, * p<0.05 and # p<0.1.

## Results

### Study population characteristics

We recruited *S. haematobium-*infected (N = 29) and -uninfected (N = 27) schoolchildren (8–16 years old) for phenotypic B cell analysis. From these, we selected 10 from each group for more extensive immunological analyses and performed follow-up studies six months later on 7 infected children treated with 3 rounds of praziquantel and 8 uninfected children. Following treatment all *S. haematobium-*infected children were infection free. As described in Table 1, there were no significant differences between the two groups in the prevalence and infection intensity of other parasitic infections such as *P. falciparum*, *A. lumbricoides*, *T. trichiura* or hookworm. Furthermore, age and gender were comparable between the two groups.

**Table 1:** Characteristics of the study population.

	*S. haematobium* infected	*S. haematobium* uninfected	p value
N	29	27	
Age in years (mean (SD))	11.36 (2.46)	11.7 (1.82)	0.887*
Male/female	18/11	14/13	0.590#
Egg counts (median (range))	11 (1–1000)	0	0.000∧
Co-infections			
*Plasmodium falciparum*	5/28	1/26	0.194#
*Ascaris lumbricoides*	5/23	5/23	1.000#
*Trichuris trichiura*	6/23	10/23	0.353#
Hookworm	3/23	1/20	0.610#

Co-infections are depicted as number of participants infected out of total number of participants tested.

* independent student's T test;

# Fisher's exact test;

∧ Mann-Whitney U test.

### Serum immunoglobulin levels

**Figure 1:**
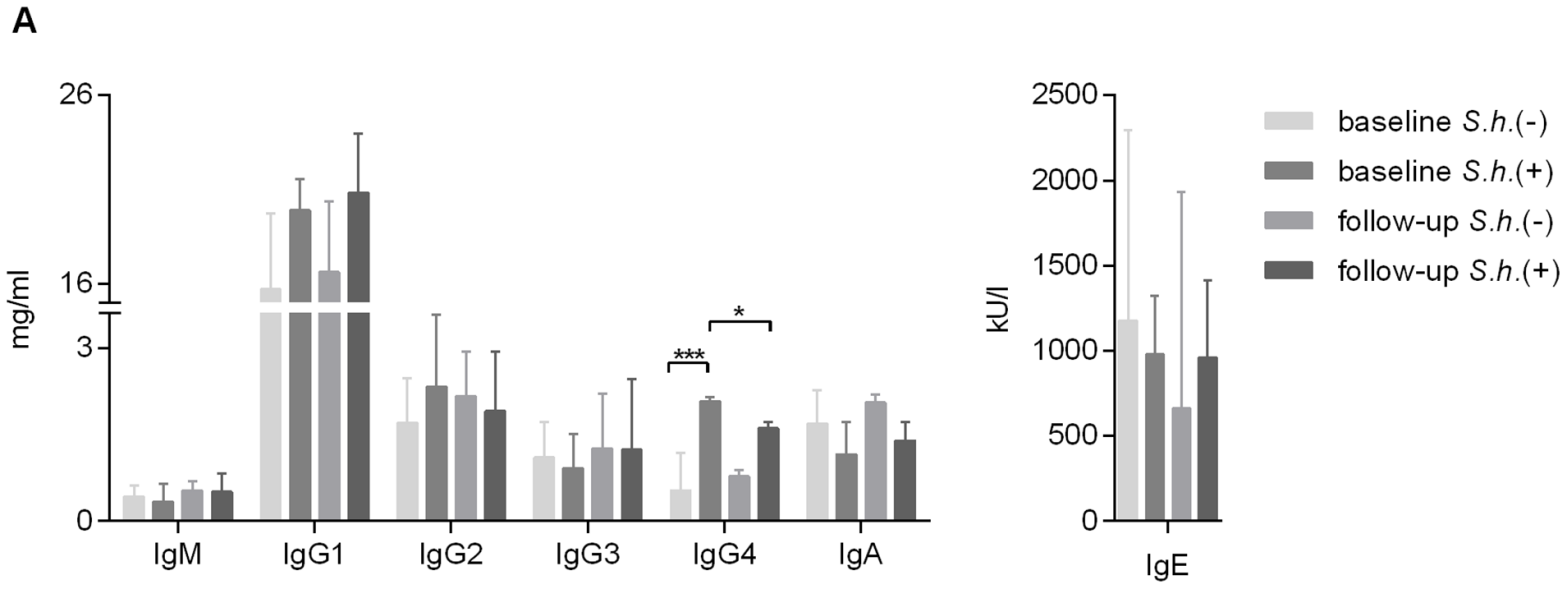
Serum immunoglobulin analysis. Serum samples were analyzed for total human IgM, IgG1, IgG2, IgG3, IgG4, IgA by Luminex and IgE by ELISA. Bars represent median with interquartile range. Number of donors in each group: baseline *S.h.* −ve n = 9, baseline *S.h.* +ve n = 10, follow-up *S.h.* −ve n = 8 and follow-up *S.h.* +ve n = 7.

In schistosomiasis, resistance is acquired slowly and it is not clear how the B cell compartment and B cell function are affected. To gain insight into global B cell function during *S. haematobium* infection we studied immunoglobulin isotypes and IgG subclasses in serum. Consistent with previously published data [43], IgG4 levels were increased in *S. haematobium*-infected compared to uninfected children and were significantly reduced following praziquantel treatment (Figure 1). No significant differences were observed in serum IgM, IgG1, IgG2, IgG3, IgA and IgE between the groups.

### B cell inflammatory cytokine response, activation and proliferation

**Figure 2:**
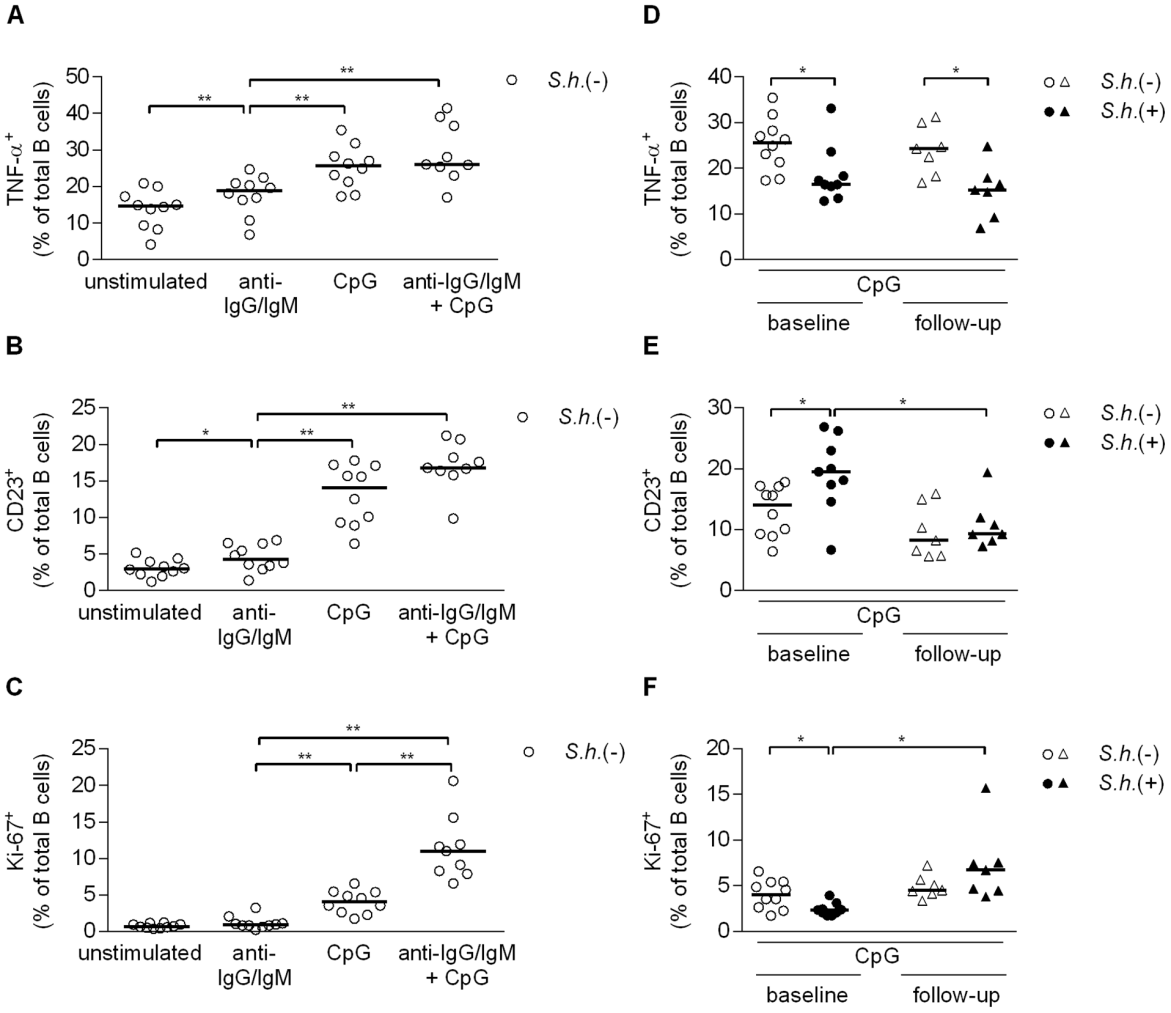
B cell inflammatory cytokine response, activation and proliferation. Total peripheral blood B cells were cultured with anti-IgG/IgM (2.5 µg/ml), CpG (5 µg/ml) or anti-IgG/IgM plus CpG for two days, restimulated with PMA/Ionomycin/LPS and BrefA and fixed. Levels of intracellular TNF-α (A), CD23 expression (B) and intracellular Ki-67 (C) were measured in *S. haematobium-*uninfected children by flow cytometry. Levels of intracellular TNF-α (D), CD23 expression (E) and intracellular Ki-67 (F) following CpG stimulation in infected and uninfected children at baseline and follow-up. Horizontal bars represent median. Number of donors in each group: baseline *S.h.* −ve n = 10, baseline *S.h.* +ve n = 9, follow-up *S.h.* −ve n = 7 and follow-up *S.h.* +ve n = 7.

To address whether B cell function was altered during *Schistosoma* infection, we measured *in vitro* cytokine responses, proliferation and activation markers of peripheral blood B cells in response to BCR (anti-IgG/IgM) and TLR9 (CpG) signaling by flow cytometry in uninfected and infected children. We first focused on responses in uninfected children. Intracellular production of the pro-inflammatory cytokine TNF-α and expression of surface CD23, an indicator of TLR activation [44], were significantly induced by BCR and TLR9 engagement; dual receptor engagement did not further increase these levels (Figure 2A, B and S1A, B). Intracellular Ki-67, a marker of proliferation, was not induced by BCR stimulation alone, but was increased following CpG stimulation, and as expected [45], dual BCR and TLR engagement was required for optimal B cell proliferation (Figure 2C and S1C). Frequencies of unstimulated B cells that produced TNF-α, expressed CD23 or were positive for Ki-67 did not differ between infected and uninfected children. As TNF-α production and CD23 expression levels were highest following CpG stimulation, with no significant enhancement when combined with anti-IgG/IgM co-stimulation, we focused on CpG stimulations for comparison between infected and uninfected children. TNF-α-producing B cell frequencies were significantly lower in infected children as compared to uninfected children (Figure 2D), and this was not upregulated upon treatment. In contrast, CD23-expressing B cell frequencies were significantly elevated (Figure 2E) and Ki-67^+^ B cells significantly reduced (Figure 2F) in the infected children; both were restored following praziquantel treatment to levels observed in the uninfected children. Baseline frequencies of CD23^+^ B cells in *ex-vivo* PBMCs did not differ between schistosome-infected children and healthy controls, 33.9% versus 40.6% respectively (p = 0.932; data not shown). Taken together, these data suggest that *Schistosoma* infection leads to alterations in B cell responses, and that some of these changes are long lasting and persist at least six months after removal of infection.

### B cell subpopulation analysis

**Figure 3:**
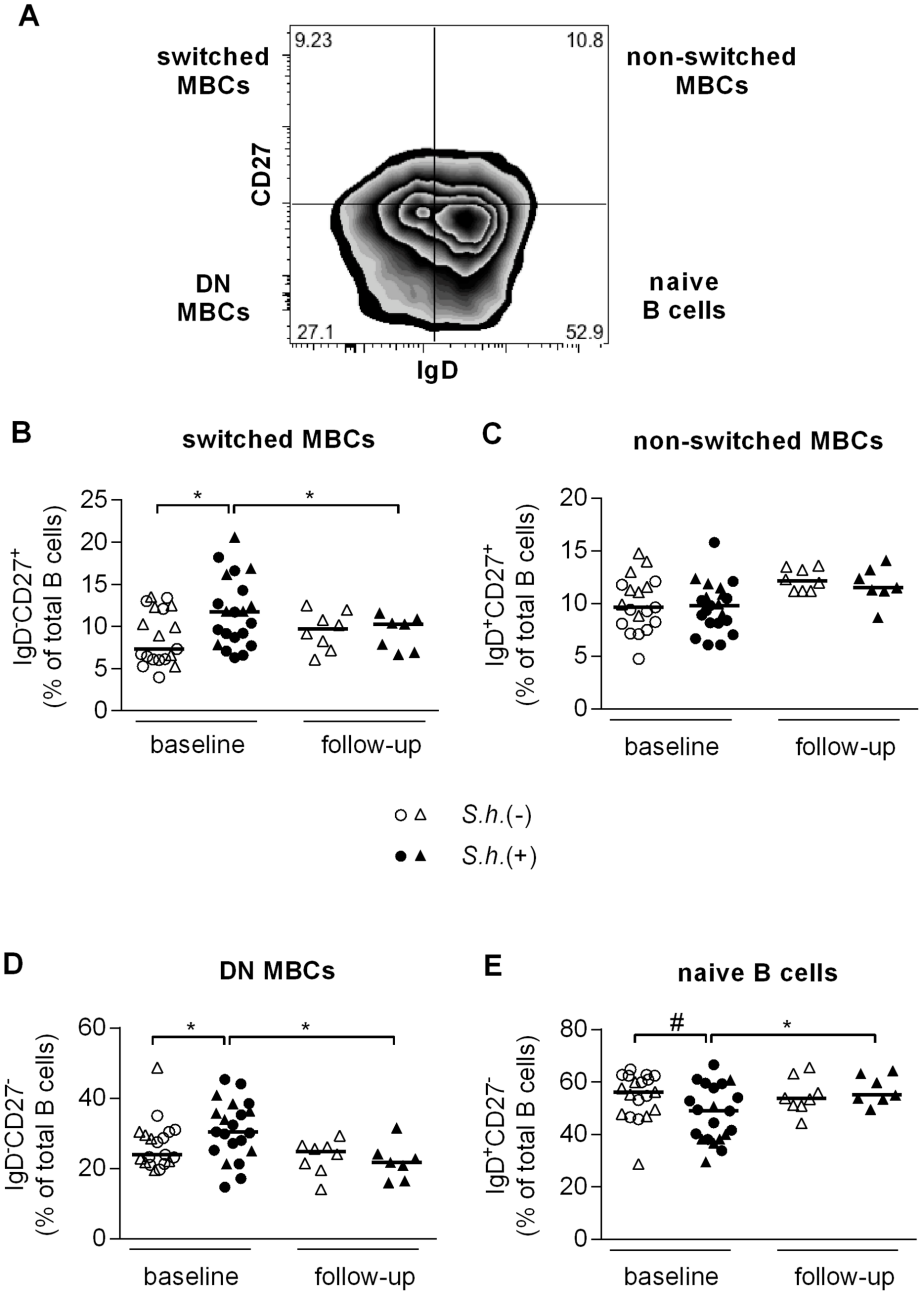
MBC analysis. PBMC were fixed and stained with B cell phenotyping markers (CD19, CD27 and IgD) and analyzed for B cell subsets by flow cytometry. B cell subset analysis was performed as shown in (A) (representative *S. haematobium-*uninfected child). Proportion of CD19-gated cells that were CD27^+^IgD^−^ (B, switched MBC), CD27^+^IgD^+^ (C, non-switched MBC), CD27^−^IgD^−^ (D, double negative MBC), and CD27^−^IgD^+^ (E, naive B cells) were determined for *S. haematobium*-infected and uninfected children at baseline and follow-up. (B, C, D, E) Horizontal bars represent median. Number of donors in each group: baseline *S.h.* −ve n = 19, baseline *S.h.* +ve n = 21, follow-up *S.h.* −ve n = 8 and follow-up *S.h.* +ve n = 7.

To further explore schistosome-induced alterations in the B cell compartment, we next compared circulating B cell subsets in peripheral blood between infected and uninfected children by flow cytometry. No statistically significant differences in the proportion of total peripheral B cells were found between schistosome-infected children and healthy controls, 15.2% versus 13.7% respectively (p = 0.776). Four distinct CD19^+^ B cell subsets can be distinguished by additional expression of CD27 and IgD [20] (Figure 3A). These are defined as naive B cells (CD27^−^IgD^+^), non-switched MBCs (CD27^+^IgD^+^) also referred to as marginal zone-like B cells, switched MBCs (CD27^+^IgD^−^), and double negative MBCs (CD27^−^IgD^−^). The proportion of switched MBCs (Figure 3B) and double negative MBCs (Figure 3D) was significantly increased in schistosome-infected children and these levels were significantly reduced to levels comparable to the uninfected control group following treatment. Concomitantly there was a trend toward a lower percentage of naive B cells (p = 0.062) (Figure 3E) in schistosome-infected children and following treatment the level of naive B cells was significantly increased. No differences were observed in the levels of non-switched MBCs (Figure 3C). Interestingly, we noted a positive correlation between total serum IgG4 levels and the percentage of switched MBCs (Spearman *r* = 0.407, p<0.05) as well as a trend with DN MBCs (Spearman *r* = 0.330, p = 0.056) and a negative correlation with naive B cells (Spearman *r* = −0.392, p<0.05).

**Figure 4:**
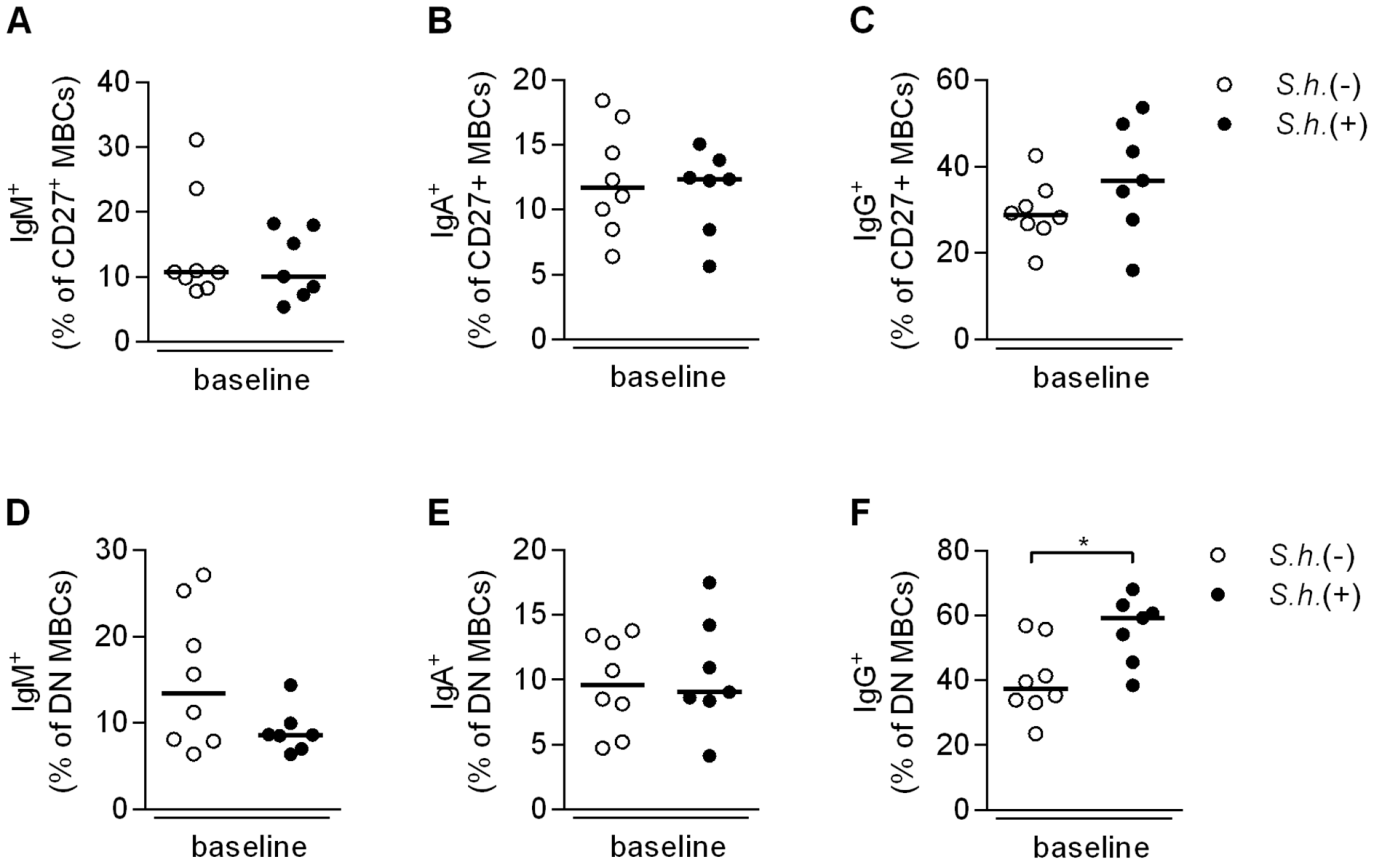
Expression of IgM, IgA and IgG on CD27^+^ and DN MBCs. PBMC were fixed and expression of IgM, IgA and IgG on CD27^+^ B cells was evaluated [46]. Proportions of CD19^+^ gated cells that were IgM^+^IgD^−^CD27^+^ (A), IgA^+^IgM^−^IgD^−^CD27^+^ (B), and IgG^+^IgM^−^IgD^−^CD27^+^ (C) were determined for *S. haematobium-*infected and uninfected children at baseline. For immunoglobulin expression on DN MBCs, PBMC were fixed and stained with B cell subset markers (CD19, IgD and CD27) and DN MBCs measured for IgM, IgA and IgG expression. Proportion of CD19^+^CD27^−^IgD^−^-gated cells that were IgM^+^ (D), IgA^+^ (E) and IgG^+^ (F) were determined for *S. haematobium-*infected and uninfected children at baseline. Horizontal bars represent median. Number of donors in each group: *S.h.* −ve n = 8 and *S.h.* +ve n = 7.

To further investigate immunoglobulin expression on B cells we evaluated an additional 8 schistosome-infected children and 8 endemic controls with an antibody panel that included a brighter CD27 antibody (Figure S2A). We found similar differences with respect to an increase in the DN MBCs (Figure S2D) and a decrease in naive B cells (Figure S2E) in schistosome-infected children. Although the switched MBCs (Figure S2B) did not differ significantly in this cohort, when grouped with the original data (from Figure 3) the overall comparison remained significant. We first evaluated immunoglobulin levels on CD27^+^ B cells [46] and found no differences in the proportion of non-switched IgM^+^ MBCs (Figure 4A), or switched IgA^+^ (Figure 4B) and IgG^+^ (Figure 4C) MBCs between schistosome-infected and -uninfected children. We next assessed immunoglobulin expression on the double negative MBCs (CD27^−^IgD^−^) and while there were no differences in the proportion of either IgM^+^ (Figure 4D) or IgA^+^ (Figure 4E) DN MBCs, the proportion of IgG^+^ DN MBCs was significantly increased in schistosome-infected children (Figure 4F). The majority of the DN MBCs were class-switched (median, 53.7%) with only 8.7% IgM^+^ cells, which may potentially be a mixture of naive and non-switched MBCs, confirming their status as memory B cells. Similar differences in immunoglobulin expression were observed on atypical MBCs (data not shown).

**Figure 5:**
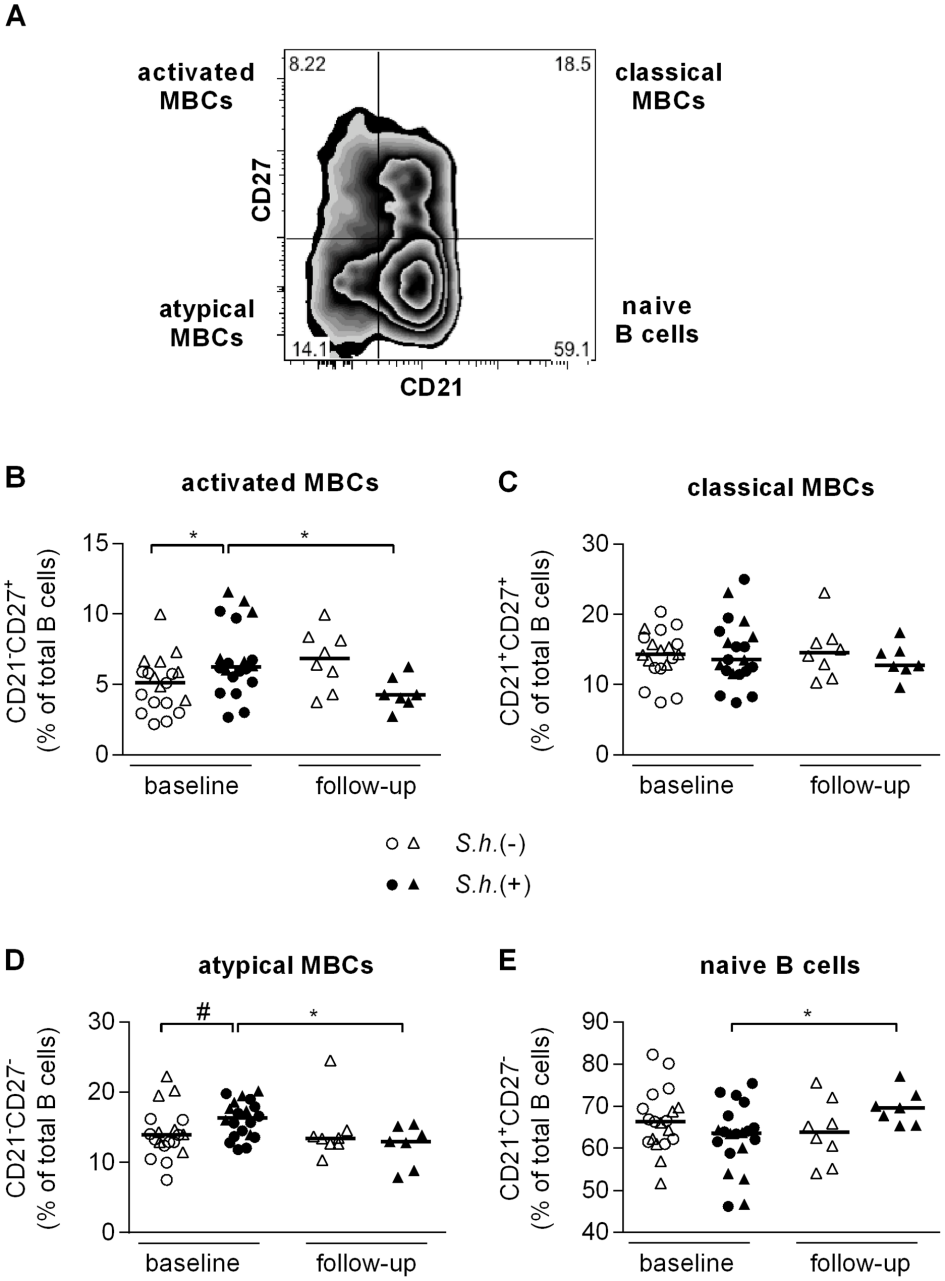
Atypical MBC analysis. PBMC were fixed and stained with B cell phenotyping markers (CD19, CD21 and CD27) and analyzed for B cell subsets by flow cytometry. B cell subset analysis was performed as shown in (A) (representative *S. haematobium-*uninfected child). Proportion of CD19-gated cells that were CD27^+^CD21^−^ (B, activated MBC), CD27^+^CD21^+^ (C, classical MBC), CD27^−^CD21^−^ (D, atypical MBC), and CD27^−^CD21^+^ (E, naive B cells) were determined for *S. haematobium-*infected and uninfected children at baseline and follow-up. (B, C, D, E) Horizontal bars represent median. Number of donors in each group: baseline *S.h.* −ve n = 19, baseline *S.h.* +ve n = 20, follow-up *S.h.* −ve n = 8 and follow-up *S.h.* +ve n = 7.

In addition to the classical characterization of the memory B cell subsets, co-staining of CD27 and CD21 [21] can be performed to identify four B cell subsets (Figure 5A): 1) naive B cells (CD27^−^CD21^+^); 2) activated MBCs (CD27^+^CD21^−^); 3) classical MBCs (CD27^+^CD21^+^) and 4) atypical MBCs (CD27^−^CD21^−^). The proportion of activated MBCs (Figure 5B) was significantly increased and there was a trend toward a higher percentage of atypical MBCs (p = 0.058) (Figure 5D) in peripheral blood of schistosome-infected children and levels of both were significantly reduced following clearance of infection. Similarly the level of naive B cells was significantly increased (Figure 5E) following treatment, while no differences were found in the proportion of classical MBCs either between the groups or at different time points (Figure 5C). While CD10, a marker of immature and germinal center B cells, was not included in these panels separate analysis showed the level of immature and germinal center B cells within our population to be ∼2.75% (median) with no differences between schistosome-infected and -uninfected children, 2.7% and 2.8% respectively (p = 0.798). To study the nature of atypical MBCs (CD27^−^CD21^−^), we determined the expression levels of HLA-DR and FCRL4, a cell surface marker that is characteristic for exhausted MBCs. In line with previous reports [23], [25], FCRL4 was expressed at significantly higher levels on atypical MBCs compared to classical MBCs (CD27^−^CD21^−^) and naive B cells (CD27^−^CD21^+^) in uninfected children (Figure S3A). Furthermore, HLA-DR expression was significantly higher on atypical MBCs and naive B cells as compared to classical MBCs (Figure S3B). Both markers were not differentially expressed between B cell subsets of uninfected and infected children (data not shown). Taken together, these data show that *Schistosoma* infection leads to changes in the distribution of peripheral B cell subsets and that praziquantel treatment leads to a reduction of various memory B cell subsets and an increase of naive B cells.

### B cell subset inflammatory cytokine response, activation and proliferation

**Figure 6:**
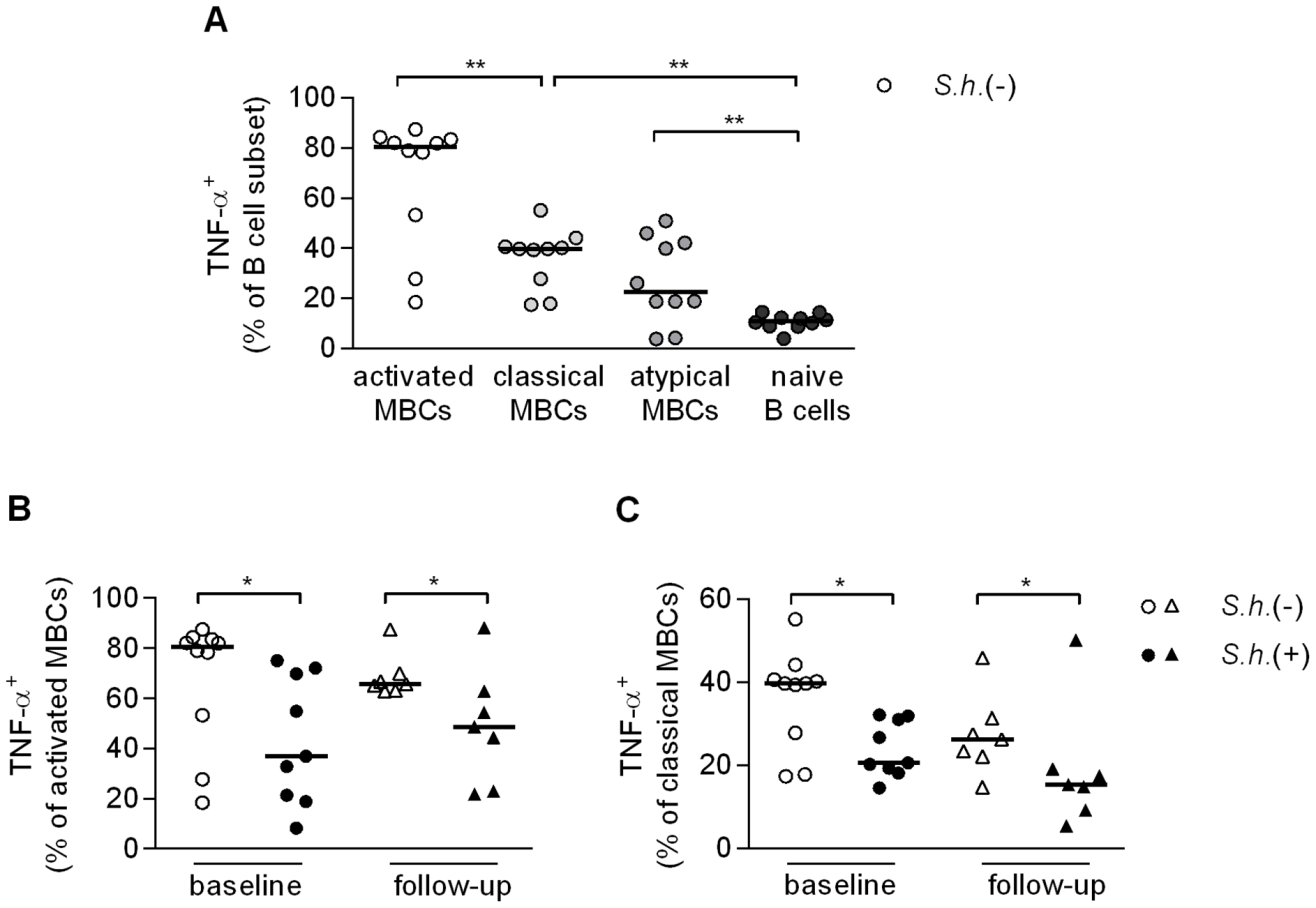
B cell subset inflammatory cytokine response, activation and proliferation. Total peripheral blood B cells were cultured with anti-IgG/IgM (2.5 µg/ml) for two days, restimulated with PMA/Ionomycin/LPS and BrefA, fixed and stained with B cell subset markers (CD10, CD19, CD21 and CD27) and levels of intracellular TNF-α was measured in *S. haematobium-*uninfected children by flow cytometry (A). Levels of intracellular TNF-α in activated MBCs (B) and classical MBCs (C) in infected and uninfected children at baseline and follow-up. Horizontal bars represent median. Number of donors in each group: baseline *S.h.* −ve n = 10, baseline *S.h.* +ve n = 9, follow-up *S.h.* −ve n = 7 and follow-up *S.h.* +ve n = 7.

To investigate whether changes in TNF-α, Ki-67 and CD23 observed in total B cells were attributed to specific B cell subsets, we extended our flow cytometry analysis to include the various B cell subsets defined by CD21 and CD27 expression, first gating on CD10^−^ B cells. Isolated B cells were stimulated with anti-IgG/IgM, CpG or a combination of the two and stained for the expression of intracellular TNF-α and Ki-67 and surface CD23. We found that CpG stimulation alone or in combination with anti-IgG/IgM resulted in significant loss of CD27 expression (data not shown); as a result it was no longer possible to differentiate the four B cell subpopulations with the same criteria as in Figure 5A. We therefore focused on anti-IgG/IgM-stimulated cells, as here we could still distinguish the four B cell populations and analyzed the responses of the various B cell subsets in uninfected children. We found very high frequencies of TNF-α-producing activated MBCs, followed by classical MBCs and atypical MBCs and observed the lowest frequencies among naive B cells (Figure 6A). When comparing the various B cell subsets for their ability to respond to BCR engagement between infected and uninfected children, we observed significantly less TNF-α^+^ activated MBCs (Figure 6B) and classical MBCs (Figure 6C) and a trend for lower levels in the naive B cells and atypical MBCs in the infected children (data not shown). These data reflect the reduced TNF-α production in the total B cell population observed earlier (Figure 2A), and likewise the levels of TNF-α following treatment were not restored to levels observed in the uninfected children for any of the subsets (Figure 6B, C). These data point at a reduced capacity of B cells from infected children to produce TNF-α in response to anti-IgG/IgM stimulation which extends to all B cell subsets studied. While classical MBCs had higher frequencies of CD23 and Ki-67 expressing cells, no differences were observed between infected and uninfected children for any of the subsets or upon treatment.

## Discussion

Although many studies have investigated the types and frequencies of various immune cell subsets, including T cells and DCs [47]–[49], little is known about the human B cell compartment during the course of *S. haematobium* infection. In the present study, we have analyzed innate and antibody-driving pathways in total B cells and different peripheral B cell subsets and compared their responses between infected and uninfected Gabonese schoolchildren. We found that *S. haematobium* infection leads to changes in B cell function as well as alterations of the total B cell compartment and these changes are not restricted to a single B cell population.

When evaluating B cell functionality, we found significantly lower frequencies of TNF-α-producing and Ki-67^+^ proliferating B cells in *S. haematobium-*infected children (Figure 2D and F). Interestingly, Ki-67^+^ B cell frequencies were restored to levels comparable to uninfected children following anti-schistosome treatment (Figure 2F), but TNF-α levels remained significantly lower (Figure 2D). The downregulation of TNF-α and B cell proliferation in *S. haematobium* positive children may be a result of immunomodulation induced by the parasite to prolong its survival. This would be in line with studies showing that TNF-α might play an important role in immunity to helminths: TNF-α production by B cells was necessary for sustained antibody production and establishment of protective immunity to *Heligmosomoides polygyrus* [50]. Moreover, B-cell derived TNF-α has been shown to enhance IFN-γ production by T cells in *Toxoplasma gondii*-infected mice [51] and it is thought that an effective Th1 response is key to natural acquisition of resistance against *Schistosoma* infection [12], [52]. Therefore, it is tempting to suggest that the long lasting reduction in B cell capacity to produce TNF-α, as demonstrated post-treatment, may in part contribute to the slow development of resistance to *Schistosoma* infection, although presence of other unknown concomitant viral or fungal infections may also play a role.

In the current study we analyzed CD23 as a TLR activation marker on B cells, however CD23 is also correlated with the development of resistance to *Schistosoma* infection [14]. Furthermore, cross-linking of CD23-bound IgE by antigen may induce cellular activation and increased IgE production [53], [54]. Repeated rounds of treatment and *S. mansoni* re-infection led to a gradual increase in CD23 expression and resistance in a cohort of Kenyan children [15]. This seems partly in contrast to our results, as the elevated CD23 levels in infected children are reduced after treatment. However in the Kenyan cohort levels of CD23 expression were evaluated in fresh whole blood samples while here we measured CD23 expression on stimulated B cells as a marker of B cell activation. Baseline levels of CD23 expression on *ex-vivo* PBMCs in our population did not differ, however CD23 levels following treatment were not measured. It would therefore be of interest to measure the dynamics of CD23 expression in our population longitudinally following multiple rounds of treatment.

Perturbations in the frequency of various B cell subsets have been demonstrated in a number of disease states [34] and here we found an increase in the switched MBCs, the double negative MBCs and activated MBCs, as well as a trend toward a higher percentage of atypical MBCs and a concomitant decrease of naive B cells in schistosome-infected children. All populations were restored to levels observed in uninfected children following treatment. It is unclear whether the increase in naive B cells following treatment is due to *de novo* generation of B cells from the bone marrow, expansion of the pre-existing naive B cell population or results from a decrease in the levels of the other subsets.

It has been shown that in HIV- [21] and malaria-infected individuals [23], [24] an exhausted/atypical memory B cell population (CD27^−^CD21^−^), was greatly expanded and that these cells were hyporesponsive and had a decreased ability to differentiate into antibody secreting cells, contributing to the diminished pathogen-specific antibody responses in infected individuals. Likewise, it has been suggested that double negative (CD27^−^IgD^−^) MBCs, which are increased in SLE, might be exhausted/terminally differentiated memory B cells [55], [56]. In chronic *S. haematobium* infection we similarly see an expansion of both double negative and atypical MBCs. The overlap between these two MBC subpopulations, their capacity to produce schistosome-specific antibodies or the exact characteristics of their ‘exhausted’ state are currently not clear.

Nevertheless it is interesting to note that schistosome-infected children carry higher frequencies of IgG^+^ double negative (CD27^−^IgD^−^) MBCs compared to uninfected children, whereas no differences are observed in the levels of IgM^+^ or IgA^+^ DN MBCs (Figure 4). Similarly, no differences were observed in total serum IgM, IgA nor IgG1, IgG2 and IgG3, whereas significant differences were only observed in IgG4 (Figure 1). Interestingly, serum levels of IgG4 were significantly decreased following treatment and correlated with a concomitant decrease in the frequency of the DN MBCs, suggesting that the increase in IgG^+^ DN MBCs may be predominantly due to an increase in IgG4-expressing B cells. As IgG4 is associated with susceptibility and IgE with resistance to *Schistosoma* infection, it would be of interest to study these isotypes on the different memory B cell populations in exposed but resistant individuals. These studies could shed further light on the various ways in which *S. haematobium* infection modulates the immune response providing further information for the design of an effective vaccine.

Although the function of double negative and atypical MBCs is not yet clear in the context of *Schistosoma* infection, it is tempting to speculate that these may be expanded as a result of the chronic nature and strong immunomodulation of *S. haematobium* infection. These memory B cells may limit the associated pathology while at the same time limiting the effectiveness of the immune response against the parasite. Indeed, a protective role against malaria infection has been proposed for atypical MBCs through regulation of the host's immune response [23], [24]. This parallels FCRL4^+^ tissue-like MBCs in lymphoid tissue, which may protect against invading pathogens directly or indirectly through the secretion of cytokines and their influence on other immune cell types [25], [26]. A recent study has highlighted the dual nature of B cells in immune responses demonstrating that the same B cells may play both a regulatory (IL-10) or pathogenic (IL-6) role depending on the signals received [57]. It would be of interest to investigate concomitantly the production of both IL-6 and IL-10 by the various MBC subsets and naive B cells to see the balance between pro- and anti-inflammatory B cell responses during *Schistosoma* infection.

As demonstrated above *S. haematobium* infection leads to significant changes in B cell function as well as alterations of the B cell compartment in peripheral blood of infected children as compared to healthy controls. Further studies are needed to define whether these changes in the frequency of the various subsets have functional consequences and what their role is in the immune response against *S. haematobium*.

## Supporting Information

Figure S1 **Gating strategy for B cell inflammatory cytokine response, activation and proliferation.** Total peripheral blood B cells were cultured with anti-IgG/IgM (2.5 µg/ml), CpG (5 µg/ml) or anti-IgG/IgM plus CpG for two days, restimulated with PMA/Ionomycin/LPS and BrefA and fixed. Levels of intracellular TNF-α (A), CD23 expression (B) and intracellular Ki-67 (C) were gated according to the gating strategy depicted in this figure (representative *S. haematobium-*uninfected child).
(TIF)

Figure S2 **MBC analysis.** PBMC were fixed and stained with B cell phenotyping markers (CD19, CD27 and IgD) and analyzed for B cell subsets by flow cytometry. B cell subset analysis was performed as shown in (A) (representative *S. haematobium-*uninfected child). Proportion of CD19-gated cells that were CD27^+^IgD^−^ (B, switched MBC), CD27^+^IgD^+^ (C, non-switched MBC), CD27^−^IgD^−^ (D, double negative MBC), and CD27^−^IgD^+^ (E, naive B cells) were determined for *S. haematobium*-infected and uninfected children at baseline. (B, C, D, E) Horizontal bars represent median. Number of donors in each group: baseline *S.h.* −ve n = 8 and *S.h.* +ve n = 8.
(TIF)

Figure S3 **Expression of FCRL4 and HLA-DR on B cell subpopulations.** PBMC were fixed and stained with B cell subset markers (CD19, CD21 and CD27) and measured for FCRL4 (A) and HLA-DR (B) expression in *S. haematobium-*uninfected children by flow cytometry. Histograms of MFI underneath are from a representative child. Horizontal bars represent median. Number of donors: baseline *S.h.* −ve n = 19.
(TIF)
