# Supplementary figures and images for: Alterations in Peripheral Blood B Cell Subsets and Dynamics of B Cell Responses during Human Schistosomiasis
Source: PLoS Negl Trop Dis. 2013 Mar 7;7(3):e2094. doi: 10.1371/journal.pntd.0002094 (PMC3591311; doi:10.1371/journal.pntd.0002094)

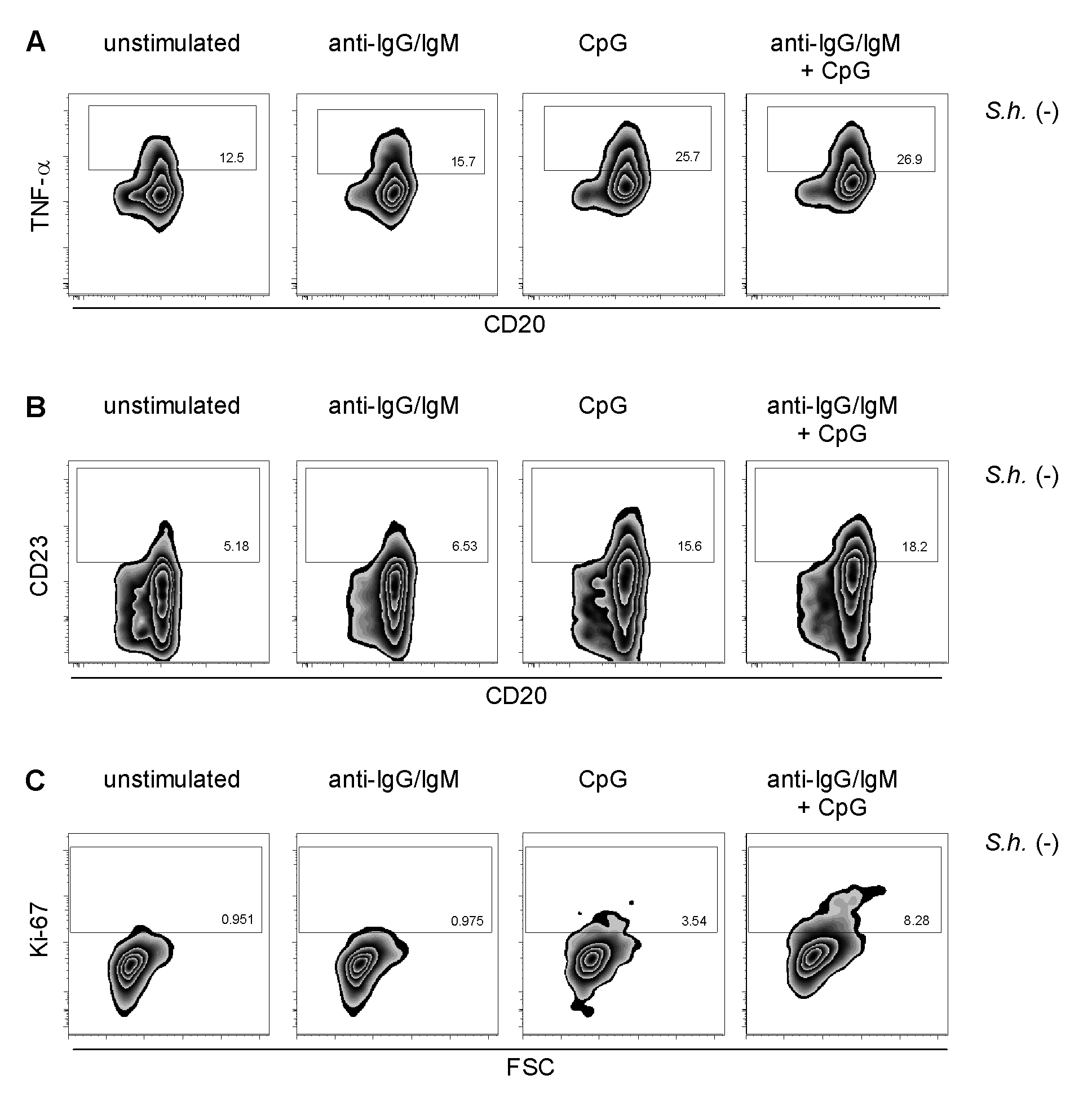

Supplement: Figure S1 — Gating strategy for B cell inflammatory cytokine response, activation and proliferation. Total peripheral blood B cells were cultured with anti-IgG/IgM (2.5 µg/ml), CpG (5 µg/ml) or anti-IgG/IgM plus CpG for two days, restimulated with PMA/Ionomycin/LPS and BrefA and fixed. Levels of intracellular TNF-α (A), CD23 expression (B) and intracellular Ki-67 (C) were gated according to the gating strategy depicted in this figure (representative S. haematobium-uninfected child). (TIF) [file pntd.0002094.s001.tif]

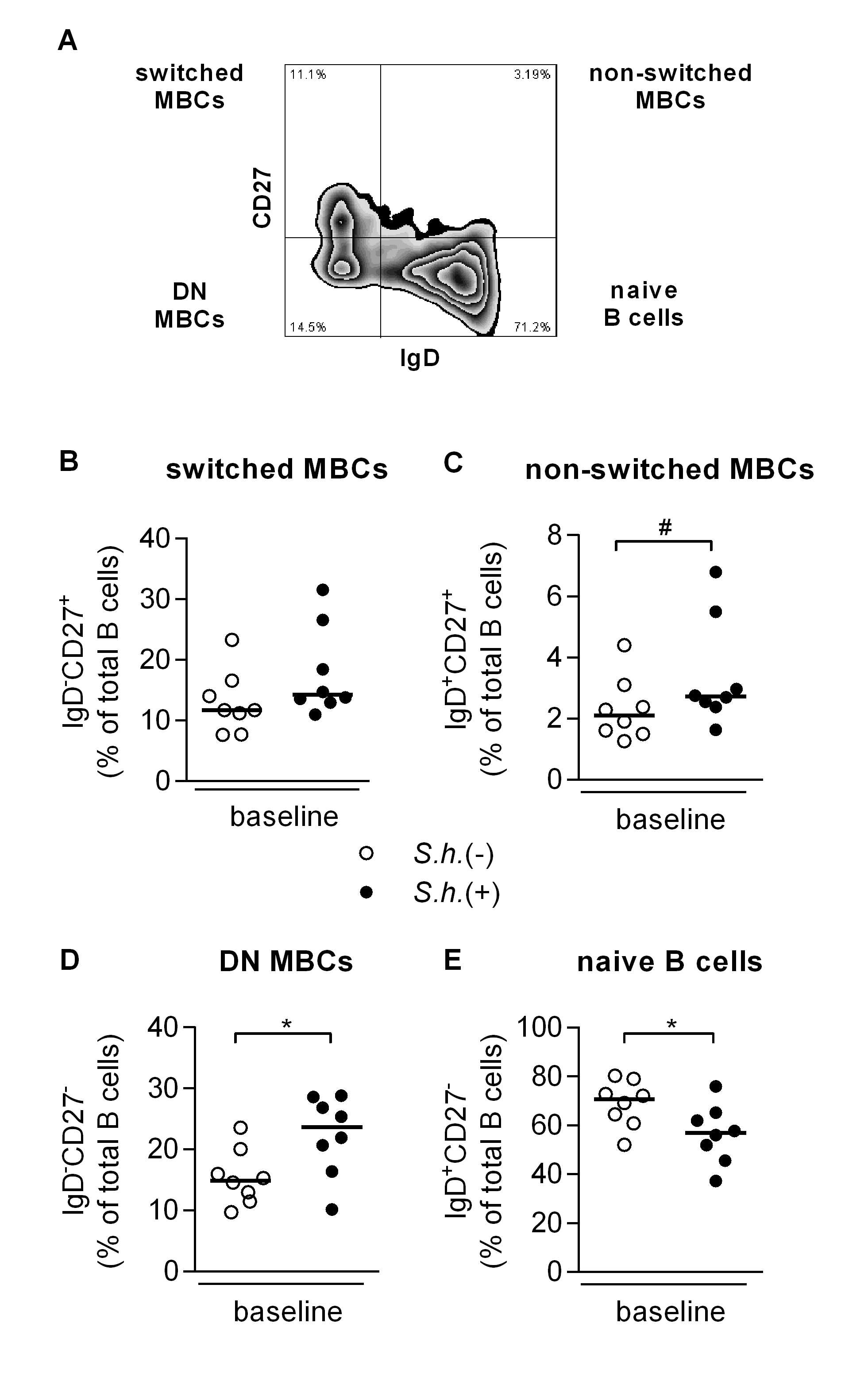

Supplement: Figure S2 — MBC analysis. PBMC were fixed and stained with B cell phenotyping markers (CD19, CD27 and IgD) and analyzed for B cell subsets by flow cytometry. B cell subset analysis was performed as shown in (A) (representative S. haematobium-uninfected child). Proportion of CD19-gated cells that were CD27+IgD− (B, switched MBC), CD27+IgD+ (C, non-switched MBC), CD27−IgD− (D, double negative MBC), and CD27−IgD+ (E, naive B cells) were determined for S. haematobium-infected and uninfected children at baseline. (B, C, D, E) Horizontal bars represent median. Number of donors in each group: baseline S.h. −ve n = 8 and S.h. +ve n = 8. (TIF) [file pntd.0002094.s002.tif]

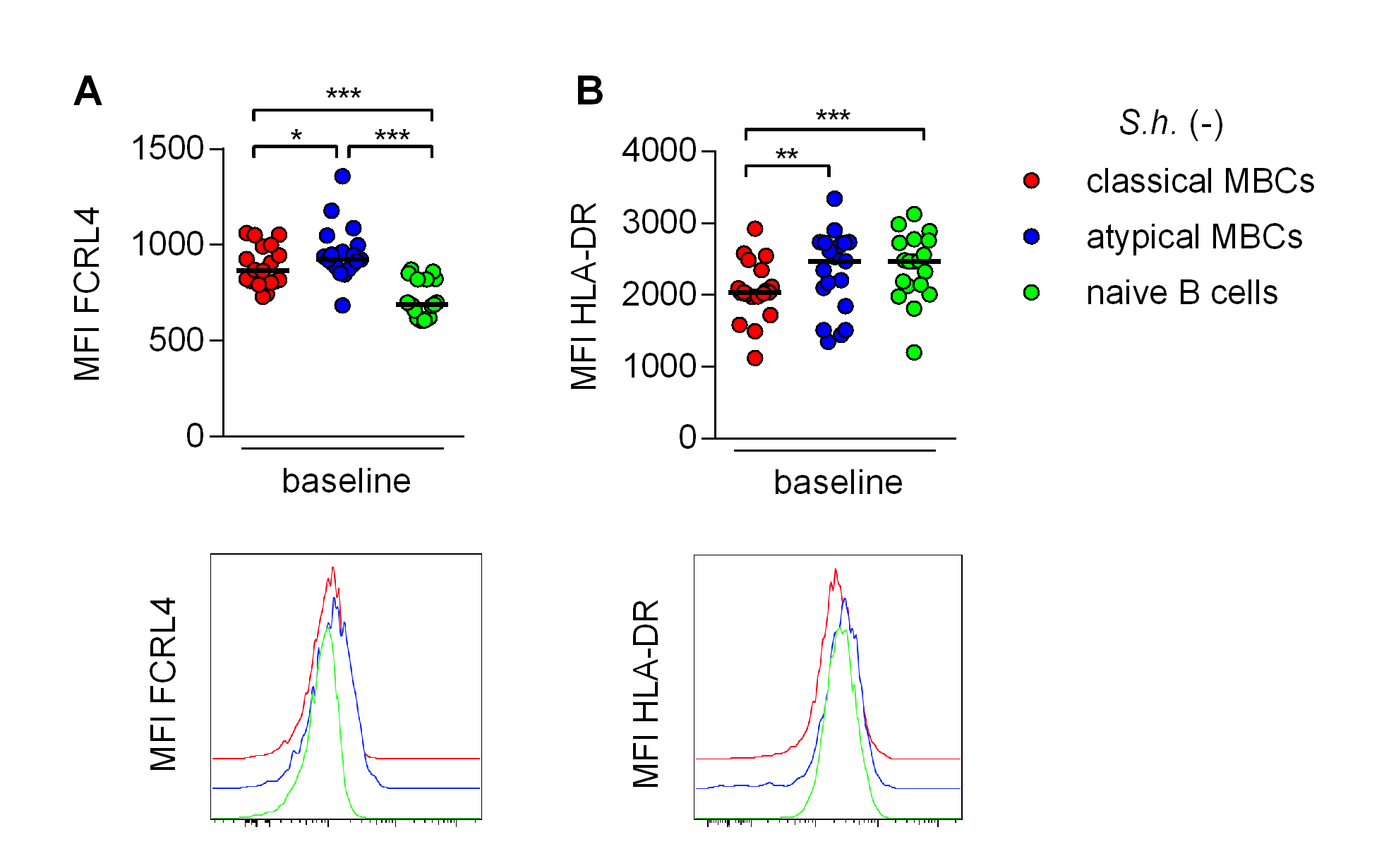

Supplement: Figure S3 — Expression of FCRL4 and HLA-DR on B cell subpopulations. PBMC were fixed and stained with B cell subset markers (CD19, CD21 and CD27) and measured for FCRL4 (A) and HLA-DR (B) expression in S. haematobium-uninfected children by flow cytometry. Histograms of MFI underneath are from a representative child. Horizontal bars represent median. Number of donors: baseline S.h. −ve n = 19. (TIF) [file pntd.0002094.s003.tif]
